# Nutmeg Extract Increases Skeletal Muscle Mass in Aging Rats Partly via IGF1-AKT-mTOR Pathway and Inhibition of Autophagy

**DOI:** 10.1155/2018/2810840

**Published:** 2018-12-17

**Authors:** Yuni Susanti Pratiwi, Ronny Lesmana, Hanna Goenawan, Nova Sylviana, Iwan Setiawan, Vita Murniati Tarawan, Keri Lestari, Rizky Abdulah, Lazuardhi Dwipa, Ambrosius Purba, Unang Supratman

**Affiliations:** ^1^Physiology Division, Department of Biomedical Sciences, Faculty of Medicine, Universitas Padjadjaran, Jatinangor 45363, Indonesia; ^2^Physiology Molecular Laboratory, Biological Activity Division, Central Laboratory, Universitas Padjadjaran, Jatinangor 45363, Indonesia; ^3^Department of Pharmacology and Clinical Pharmacy, Faculty of Pharmacy, Universitas Padjadjaran, Jatinangor 45363, Indonesia; ^4^Geriatric Subdivision, Department of Internal Medicine, Faculty of Medicine-Hasan Sadikin Hospital, Universitas Padjadjaran, Bandung 40161, Indonesia; ^5^Department of Chemistry, Faculty of Mathematics and Natural Sciences, Universitas Padjadjaran, Jatinangor 45363, Indonesia

## Abstract

The sarcopenic phenotype is characterized by a reduction of muscle mass, a shift in fiber-type distribution, and reduced satellite cell regeneration. Sarcopenia is still a major challenge to healthy aging. Traditional Indonesian societies in Sulawesi island have been using nutmeg for maintaining health condition during aging. Interestingly, nutmeg has been known to stimulate peroxisome proliferator activated receptors *γ* (PPAR*γ*) which may contribute to myogenesis process in cardiac muscle. There is limited information about the role of nutmeg extract into physiological health benefit during aging especially myogenesis process in skeletal muscle. In the present study, we want to explore the potential effect of nutmeg in preserving skeletal muscle mass of aging rats. Aging rats, 80 weeks old, were divided into two groups (control and nutmeg). Nutmeg extract was administered for 12 weeks by gavaging. After treatment, rats were anaesthesized, then soleus and gastrocnemius muscles were collected, weighted, frozen using liquid nitrogen, and stored at -80°C until use. We observed phenomenon that nutmeg increased a little but significant food consumption on week 12, but significant decrease in body weight on weeks 10 and 12 unexpectedly increased significantly in soleus muscle weight (*p<0.05*). Nutmeg extract increased significantly gene expression of myogenic differentiation (MyoD), paired box 7 (Pax7), myogenin, myosin heavy chain I (MHC I), and insulin-like growth factor I (*p<0.01*) in soleus muscle. Furthermore, nutmeg increased serine/threonine kinase (AKT) protein levels and activation of mammalian target of rapamycin (mTOR), inhibited autophagy activity, and stimulated or at least preserved muscle mass during aging. Taken together, nutmeg extract may increase muscle mass or prevent decrease of muscle wasting in soleus muscle by partly stimulating myogenesis, regeneration process, and preserving muscle mass via IGF-AKT-mTOR pathway leading to inhibition of autophagy activity during aging. This finding may reveal the potential nutmeg benefits as alternative supplement for preserving skeletal muscle mass and preventing sarcopenia in elderly.

## 1. Introduction

Aging in human and animal model is associated with a progressive loss of muscle mass and strength, which results in a condition known as sarcopenia. Sarcopenia is a geriatric syndrome with progressive loss of skeletal muscle mass and function. It is characterized by atrophy of type II muscle fiber and reduction in muscle fiber satellite cells with aging [1]. Reduction of the satellite cells declines regenerative capacity that caused loss in type IIb fibers skeletal muscle and to lesser extent type I fibers [2]. The loss of muscle mass and function is caused by a series of complex factors including the accumulation of denatured, mis-folded, cross-linked, or aggregated molecules and has deleterious effects on the quantity and quality of muscle [3, 4]. There are many intrinsic factors correlated with sarcopenia as well as systemic inflammation, high glucocorticoids levels, increased mitochondrial abnormality, excessive apoptosis, and decreased satellite cell activity [5, 6].

Aging is also associated with changes in hormone levels, such as those of growth hormone (GH), insulin-like growth factor I (IGF1), insulin, androgen, estrogen, and corticosteroid, which affect the anabolic and catabolic conditions for muscle protein metabolism [7]. Lower level of IGF1 and insulin resistance lead to decrease protein synthesis [8]. Previous study using weight-matched genetically obese-leptin receptor deficient mice (db/db mice) showed that insulin resistance causes muscle wasting by mechanism that involves supression of phosphatidylinositol 3-kinase (PI3K) serine/threonine kinase (AKT) signalling causing muscle protein degradation [9]. Preserving the skeletal muscle mass needs the equilibrium between protein synthesis and protein breakdown. Disturbance of balance between protein synthesis and breakdown was observed in sarcopenic condition. Lower rates of muscle protein synthesis lead to muscle wasting in the elderly [10] and elevated basal rates of muscle protein breakdown [11]. In addition, some hormones lik testosterone, insulin, and IGF1 are potent activators of the AKT pathway that can lead to increased muscle protein synthesis and decreased protein degradation by inhibiting FoxO members of the class O of forkhead box transcription factors [12, 13]. On the other hand, another hormone, thyroid hormone, has opposite role in autophagy; it induces autophagy activity in soleus muscle which is essential for mitochondrial biogenesis and potential muscle fiber shifting [14].

Physical exercise and nutrient anabolic upregulate muscle mass growth through IGF1 and PI3K and their downstream mediators AKT and mammalian target of rapamycin (mTOR), as the core components of PI3K/AKT/mTOR signalling pathway [15, 16]. Yet, depending on the other factors that may also overlapping by complex interaction, physical exercise and nutrient anabolic alone are less effective in restoring aging muscle mass. In some specific clinical condition such as stroke and paralyzed, it is really difficult to persuade sarcopenic patients to follow an exercise programme; therefore combination between anabolic nutrients, physical exercise, supplement, and hormone treatment may help us to find best solution for preventing sarcopenia in specific conditions.

As a tropical country, Indonesia has a rich diversity of flora (herbal plant) and great potential health benefit effects in its active compound. Some herbal plant has rich content of active compound like catechin, resveratrol, and curcumin which showed potential effects on muscle regeneration in skeletal muscle. Keri Lestari et al. had showed that nutmeg extract has peroxisome proliferator activated receptors *γ* (PPAR*γ*) ligand activity [17]. The nuclear receptor PPAR*γ* plays a key role in regulating whole body glucose homeostasis and insulin sensitivity. Although this gene is expressed mostly in adipose, it is also present at lower level in many tissues, including cardiac and skeletal muscle. PPAR*γ* also had reported play role in cardiomyogenesis which showed a possibility of controlling its similar action in skeletal muscle [18, 19].

In the present study, we want to explore potential effect of nutmeg extract for preserving muscle mass using aging rats' model. Our study showed that nutmeg induced IGF1-AKT-mTOR pathway leads to autophagy activity inhibition and may increase or at least preserve skeletal muscle mass in rats.

## 2. Materials and Methods

### 2.1. Nutmeg Extract

The nutmeg extract used in this study is free safrole and myristicin nutmeg extract. The dried seeds of* Myristica fragrance* were collected during dry season from Maluku and West Java Island. Thirty kilograms of powdered seed was extracted with 225 l ethanol 95% at room temperature using a pilot scale extractor with a circulator rate of 150-200 rpm for 30 minutes. The extract was evaporated at a temperature range of 40-60°C and pressure range of 400-500 mmHg.

### 2.2. Food Intake and Body Weight

Preweighted food was provided in standard steel hoppers. After 24 hour, rats were briefly removed from their cages and weighed, and the amount of food remaining, including any on the bottom of the cages or any that spilled onto plastic sheets placed under each cage, was recorded. Food intake was calculated as the weight (in grams) of food provided less that recovered. We counted the weight of food intake and body weight and analyzed it weekly by comparing mean total food intake and mean total of body weight from both groups.

### 2.3. Analysis of Safrole

The concentration of safrole was analyzed with the high-performance liquid chromatography system (Waters 2998). The analysis was performed using C18 columns (LiChroCART 250-4 LiChrosphere 100 18e (5*μ*m) (Merck)). The mobile phase consisted of a 27:73 (v/v) mixture of water (component A) and methanol (component B). Chromatographic separation was carried out at room temperature (25°C, maintained with air conditioning) and the injection volume was 20 *μ*l. Data which were collected with a photodiode array detector wavelength of 200-400 nm were used for analysis.

### 2.4. Safrole Removal

900-gram powdered extract was prepared for safrole removal using a pilot scale column chromatography. The column consists of upper and lower parts containing mesh size of 70-230 silica as the stationary phase. For removal step, 130 L of a mixture of n-hexane: ethyl acetate (9:1) was used as mobile phase at a flow rate of 6 L/min and elutes were discarded. The residue was then eluted by 100 L methanol, and all of elutes were collected, evaporated, and analyzed for safrole content.

### 2.5. Animals

Animal handling, maintenance, and euthanasia procedures were performed after approved by the Ethics Committee, Faculty of Medicine, Universitas Padjadjaran.

### 2.6. Aging Rat Model

Male, 20 Wistar rats, aged 80 weeks (Supplementary Figure 1), were bred in the Animal Facility of PT Bio Farma, Indonesia. Rats were kept at 24°C under a 12-hour light, 12-hour dark cycle (light on, 6 am to 6 pm), and 55% relative humidity, with food and water ad libitum for 80 weeks in Animal Laboratory, Physiology Division, Faculty of Medicine, Universitas Padjadjaran.

### 2.7. Animal Treatment

Twenty male Wistar male rats, 80 weeks old, with weight around 450 grams were randomly divided into control and treatment group. Nutmeg extract was given to the treatment group for 12 weeks, and water was given to the control group by gavage. The dose administered to the treatment group was a conversion of the human dose (300 mg/day) of 8.1 mg/day/kg body weight. The specific dose was counted for each rat. Nutmeg and water were given daily every morning at the same time for 12 weeks. The negative control group was given PGA 2%. The rat was sacrificed using isoflurane anaesthesia, then soleus and gastrocnemius muscle were removed, weighed, and rapidly frozen in liquid nitrogen and stored at −80°C until use.

### 2.8. RNA Extraction and Quantitative RT-PCR

Total RNA from muscle tissue was isolated with Trizol reagent from Invitrogen, USA, and used according to the manufacturer's instructions. Onestep Polymerase Chain Reaction** (**PCR) kit (Bioline, USA) was use in this study. Specific primers for MyoD, Pax7, IGF1, Myogenin, MHC I, and *β*-actin as internal controls were used. Primer sequences and annealing temperatures are shown in Supplementary Table 1. The PCR results for each sample were normalized with *β*-actin mRNA levels as an internal control.

### 2.9. Western Blot Analysis

The dissected soleus muscle was weighted, homogenised in lysis buffer containing 10 mM Tris-HCl (pH 7.8), 150 mM NaCl, 1 mM EDTA, 1% Nonidet P-40, and protease inhibitors. After centrifugation, protein samples were heat denatured at 96°C for 5 minutes. Samples (10 *μ*g/lane) were separated by SDS-PAGE and were then transferred to a nitrocellulose membrane (GE Healthcare) for 1 hour at room temperature and blocked overnight at 4°C in 2% blocking reagent (GE Healthcare) in Tris-buffered saline buffer with 0.1% Tween 20. Immunoblotting was performed using a mouse monoclonal anti-mTOR (#2972); AKT (#9272), p-mTOR (#2971), LC3 (#12741), p62 (#5114), and Glyceraldehyde-3-Phosphate Dehydrogenase (GAPDH) thermo scientific AM4300 were purchased from Cell Signalling Co., Ltd.) with dilution 1:1000. The signals were developed using enhanced chemiluminescence reagent (GE Healthcare) and imaged (LI-COR C-DiGit Chemiluminescence Western Blot Scanner). The band intensities were determined using ImageJ Software (NIH). Blots were stripped using stripping buffer from thermo scientific according to manufacturer protocols and reprobed using with an antiGAPDH as internal control to monitor the level of protein.

### 2.10. Statistical Analysis

Data were analyzed with One-Way Analyze of Variance (ANOVA) test using SPSS V.13. Statistical significance was designated at* p <0.05*. Data are expressed as mean ± standard error minimum (SEM).

## 3. Results

### 3.1. Food Consumption and Body Weight between Two Groups of Aged Rats

**Figure 1:**
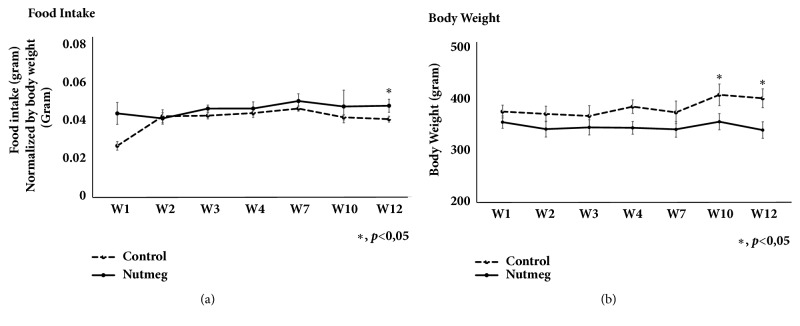
Nutmeg group has lower body weight with no different of amount food consumption. (a) Observation from weekly food consumption amount is not statistically different between nutmeg and control group; only in week 10 nutmeg showed increase of food consumption. (b) Nutmeg group rats have significantly lower body weight compared to control group. Data represent mean ± SEM of experiments. *∗*p <0.05 compared with control group.

The observation of food consumption and body weight was done weekly. We observed there is no significant difference in food consumption in week 12; however there is slight increased food consumption (10%) in nutmeg group compared to control group (Figure 1(a)). Body weight was decreased significantly 10% in nutmeg group (Figure 1(b)) in weeks 10 and 12. Food consumption and body weight were obtained to observe possible PPAR*γ* activity effect from nutmeg that may alter general metabolism condition in aging rat models.

### 3.2. Nutmeg Treatment Increased Soleus Muscle Weight but Not in Gastrocnemius Muscle Weight

**Figure 2:**
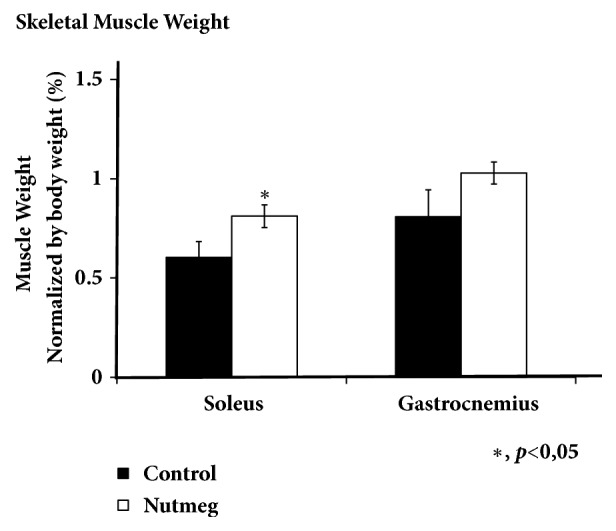
Nutmeg treatment increases soleus significantly muscle mass and not in gastrocnemius muscle. Data represent ratio in mean ± SEM of experiment. *∗*p <0.05 compared with control group. After 12 weeks of nutmeg treatment, soleus and gastrocnemius muscle from both groups were weighed.

Soleus muscle (type I muscle) and gastrocnemius muscle (mix of type I and II muscle) were collected, weighed, and normalized then counted as ratio. Soleus and gastrocnemius muscle were cleanly removed, tendon to tendon, and weighed from both groups. Soleus muscle weight was significantly increased by 1, 34 times higher compared to control group (*p<0.05*). We also observed gastrocnemius muscle mass was increased by 1, 27 times higher compared to control; however its increasement was not significant (Figure 2). We analyze the muscle weight to explore whether body weight difference affected the muscle weight.

### 3.3. Gene Regulating Muscle Satellite Cell, Proliferation, Regeneration, Type of Fiber, and IGF1 Was Increased in Soleus Muscle with Nutmeg Treatment

**Figure 3:**
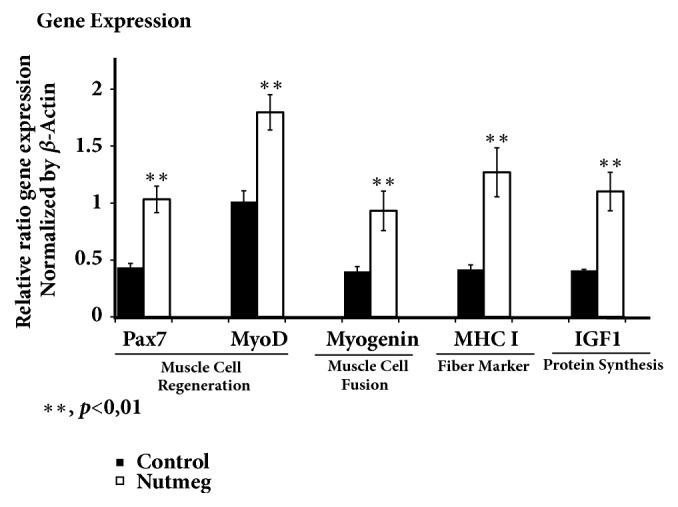
Nutmeg increases gene expression of Pax7, MyoD, myogenin, MHCI, and IGF1 in soleus muscle. Data represent ratio in mean ± SEM of experiment. *∗∗*p <0.01 compared with control group. Increasing Pax7 and MyoD gene expression shows that there is muscle growth and regeneration occurring after 12-week nutmeg extract treatment on aging rats. Increasing another gene related to muscle cell fusion, myogenin (p <0.01) supports another result that nutmeg stimulates muscle growth. To more support the result and the pathway involving, observation from gene related to fiber marker MHC1 also increased (p<0.01) and also genes have pivotal role in protein synthesis muscle hypertrophy IGF1 (p<0.01).

Analyzing gene expression of myogenic differentiation (MyoD) and paired box 7 (Pax7), myogenin, myosin heavy chain (MHC I), and IGF1 may correlate with soleus muscle weight increase in nutmeg group. Nutmeg group induced significantly expression of Pax7, MyoD, and myogenin gene compared to control groups (Figure 3) that play role in myogenesis process correlated with increased muscle weight. In addition, IGF1 which has role in protein synthesis increased in nutmeg group. Surprisingly, MHC1 gene important for formation of fiber type II was also stimulated by nutmeg treatment (Figure 3).

### 3.4. AKT-mTOR-Autophagy Pathway (AKT-mTOR Stimulated, Autophagy Blocked) in Soleus Muscle with Nutmeg Treatment

**Figure 4:**
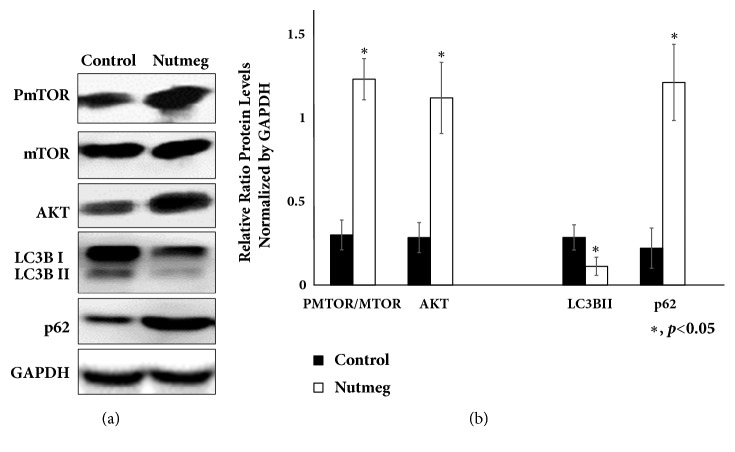
Nutmeg stimulates IGF1 signalling pathway and induced AKT and mTOR signalling. This induction stimulated inhibition of autophagy activity in soleus muscle. Representative immunoblot was shown (a) and densitometric quantification was shown in the graph normalized by GAPDH. Data represent ratio in mean ± SEM of experiment. *∗*p <0.05 compared with control group. Increasing level of AKT-mTOR supports that nutmeg stimulates muscle protein synthesis most probably through activation of IGF1, AKT, and mTOR pathway. The protein level of p62 and LC3BII shows that activation of IGF1, AKT, and mTOR pathway leads to inhibition of autophagy process.

Sarcopenic muscle has functional defect in autophagy-dependent signalling. Furthermore, autophagy may also involve in the balance of protein synthesis and protein degradation in myogenesis. We observed AKT-mTOR protein was stimulated by nutmeg treatment important for protein synthesis as start point of IGF1-AKT-mTOR signalling pathway. This result is consistent with the upregulation of IGF1 gene expression. The activation of AKT signalling by IGF1 blocks FoxO-dependent transcription for the inhibition of protein degradation. mTOR also plays a crucial role in the regulation of protein translation. mTOR has been shown to inhibit autophagy initiation. Increased an autophagy mediator (p62) and decreased microtubule-associated protein light chain 3 (LC3BII) in nutmeg group indicate inhibition of autophagy (Figure 4).

## 4. Discussion

Aging causes skeletal muscle mass decrease. Aging-related apoptosis in specific muscle fibers is the characteristic consequence of sarcopenia. Mitochondrial-mediated apoptosis has been postulated as one of the mechanisms associated with muscle fiber loss. In the aging process there is a 10-40% decrease in type II muscle fibers but the type I muscle fibers are generally unaffected. This variability in contractile properties is achieved mainly by diversification in the motor protein myosin heavy chain (MHC) where different isoforms are encoded by distinct genes. Types I, IIa, Iix, and IIb in respective order in increasing ATPase activity are the four predominantly expressed MHC isoforms. Myosin-1 also known as striated muscle myosin heavy chain 1 is a protein encoded by MYH1 gene that is also highly expressed in fast type IIX/D muscle fibers and encodes class II myosin. Myosin heavy chain I (MHC 1) is also known as MYHa, MYH1, MyHC-2X, and MyHC-2X/D. Type 2X muscle fibers are expressed by MYH1 gene in adult muscle [20].

The composition of muscle fibers may change as a response of various external stimuli. Type I is more susceptible to inactivity and denervation-induced atrophy, whereas type II is more affected by aging, diabetes, cancer, and metabolic syndrome. During the early stage of sarcopenia, this loss can be attributed to type II and fibers shifting from type II to type I. Although sarcopenia is widely considered to preferentially impact type II muscles, another study also found that relative protection to the slow twitch muscle form age-related atrophy is present until middle age with a great degree of atrophy present thereafter. Slow twitch soleus muscles undergo large phenotypic alterations in very old age [3, 21]. Our observation showed that nutmeg treatment has increased muscle mass, both type I fibers muscle-soleus and mix of type I and II muscle fibers-gastrocnemius (Figure 2) even though nutmeg group has lower body weight after 12 weeks of nutmeg treatment (Figure 1(b)). Soleus muscle is a typical muscle composed mainly with type I fibers (more than 90%) which allow prolonged and steady contraction. The gastrocnemius muscle is composed of both type I and II muscle fibers which ensure rapid and steady movement [22]. Those both muscle fiber types can be cleanly taken from hind limb and ideally used as muscle weight ratio countification. Increase of skeletal muscle weight ratio after nutmeg treatment showed that it may increase protein synthesis or prevent excessive protein breakdown in type I and mixed types I-II that usually occurs in aging. We observed a novel potential function of nutmeg extract to induce or at least preserve muscle mass in aging rat especially in soleus muscle (Figure 2), although having lower body weight (Figure 1(b)). We also found that nutmeg extract may induce food consumption in week 12 (Figure 1(a)); this could be dopamine 1 A stimulation which is responsible in appetite modulation [23].

In aging skeletal muscle, both decreasing protein synthesis and increasing protein breakdown are important events for atrophy. The equilibrium between protein synthesis and protein breakdown is required for maintaining muscle mass and muscle function and plays a pivotal role in muscle regeneration capacity. Muscle mass regeneration requires increase in protein synthesis and decrease in protein breakdown. However, the equilibrium is not easily achieved especially in the elderly due to a lot of physiological processes that altered during aging [12, 24]. Nutmeg extract previously found has a positive effect on insulin sensitivity as PPAR*γ* agonist [17]. PPAR*γ* activation enhances insulin sensitization in skeletal muscle and increases glucose metabolism and adipogenesis in white and brown fat tissue [18, 25], cause-effect relationship between insulin resistance and the stimulation of muscle protein breakdown. PPAR*γ* selective agonist agent- rosiglitazone improves the insulin resistance and concomitantly also showed decrease in proteasome proteolytic activity [9].

Insulin resistance alters some growth factor signalling cascades and can variably inhibit muscle regeneration. PPAR*γ* may indirectly affect IGF1 as in function in regulating glucose metabolic levels in skeletal muscle. As a central pathway in the regulation of muscle metabolism, IGF1 signalling also can regulate the transcription of AKT and FoxO followed by upregulating of mTOR signalling in skeletal muscle. The binding of insulin and IGF1 with membrane receptors can activate AKT/mTOR-mediated signal transduction and inhibit proteolysis, finally leading to the growth of skeletal muscle [3, 16, 26]. Secretion of growth hormone such as IGF1 declines continuously to very low level in those elderly age. This decrease is associated with decreased protein synthesis and increased adipocyte infiltration. We observed that nutmeg increases gene expression of IGF1 (Figure 3) together with protein AKT and mTOR (Figure 4). Nutmeg may have an effect to increase protein synthesis especially in type II fibers soleus muscle of aging rats due to MHC1 gene stimulation.

Nutmeg increases gene expression of IGF1 together with Pax7, MyoD, MHC I, and myogenin significantly in soleus muscle (Figure 3). Increased gene of Pax7 stimulation may contribute to the increase of regenerative potential of aged muscle, where the decrease of Pax7 pool correlated with sarcopenia. MyoD is considered the myogenic master gene as its activity can trigger the entire myogenic program [27]. Further, Pax7 cointeract with MyoD and myogenin showed that differentiation of myoblast may occur in aged rats model (Figure 3). Previous utilisation of unique cell surface markers to identify satellite cell proliferation and differentiation as well as Pax7 and MyoD has provided evidence to show its importance in muscle growth and repair as well as in the process of adaptation to stress including exercise and aging. Some studies using different Pax7 ablation strategies in mouse muscles have clearly shown that satellite cells are indispensable for muscle regeneration. One of the potential mechanisms for the reduction of skeletal muscle mass during aging is the failure of satellite cells to replace and repair damaged muscle fibers [28, 29]. The controversy concerns the capacity of skeletal muscle precursor cells (widely referred to as myogenic stem cells and satellite cells) especially in response to exercise, supplementation, and protein intake [3, 30, 31]. Increase of satellite cells and MHCI gene expression after nutmeg consumption may be a direct effect since satellite cells can be modulated by some factors such as metabolic regulation [27], mitochondrial function [26], and exercise regulation [24] and also via nutraceutical like nutmeg especially in type I muscle fibers.

The result from this study showed that nutmeg increases mTOR and AKT together with inhibition of autophagy (Figure 4). Alteration of autophagy activity can be observed in both skeletal and cardiac muscles during aging, although the mechanism for the impairment in autophagy appears to be different between these tissues and is still unclear [32]. As an essential homeostasis mechanism, autophagy is proposed to be a critical physiological process and proteolytic system for degradation of cytoplasmic constituents including protein aggregates and organelles. In aging process, dysregulated autophagy is attributed to the apparent aging-related accumulation of damaged cellular components such as defective mitochondria, which results in more reactive oxygen species (ROS) [12]. Autophagy can promote either cell survival or cell death depending on the circumstances. Both excessive and defective autophagy will be highly correlated with the loss of skeletal muscle. The deficiency of basal autophagy can result in the abnormal aggregation of mis-folded protein, where the excessive autophagy also can cause cellular stress and induce the loss of skeletal muscle due to increased protein breakdown. Sarcopenia is a process in which skeletal muscle fibers gradually lose the capacity to adapt the changing environments and present the accumulation of damaged products due to overall decrease of protein degradation process including autophagy pathway [12, 24].

Increase of p62 and decrease of autophagosome marker Atg8/LC3BII after 12 weeks nutmeg treatment showed that nutmeg extract inhibits autophagy process in soleus muscle (Figure 4). High level of p62 protein usually indicates accumulation of sequestered protein target of autophagy process [33]. Inhibition of autophagy results in accumulation of both p62 and ubiquitinated proteins in skeletal muscle. Autophagy role and modulation in skeletal muscle occurring with several physiological and pathological conditions such as fasting, atrophy, and exercise have been investigated, but the functional relationship between autophagy and cell metabolism during the differentiation or myogenesis process remains to be investigated further. Our findings showed inhibition of autophagy process is involved in maintaining muscle mass. Even though autophagy is required for maintaining biogenesis mitochondria in skeletal muscle function, many factors can alter its homeostasis activity level of autophagy. Induction or inhibition of autophagy can be positively contributes to physiological muscle function and mass. Effects of aging on autophagy alteration in skeletal muscles have no consistent pattern. As example, mice with inhibited autophagy TSC-1 deficient mice and ATG7 KO mice showing a reduction in myofiber size demonstrate that autophagy is required to maintain muscle mass [34].

**Figure 5:**
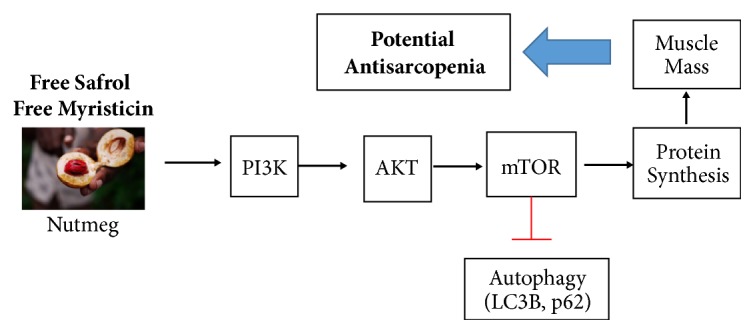
Proposed scheme of nutmeg action in soleus muscle. Activation of phosphatidylinositol 3-kinase (PI3K) pathway can promote skeletal muscle hypertrophy and increase skeletal muscle mass. This pathway's effect on skeletal muscle has implicated most prominently downstream of IGF1 signalling. IGF1 can lead to muscle hypertrophy coming predominantly through its ability to activate the PI3K/AKT signalling pathway. AKT is a serine-threonine protein kinase that can induce protein synthesis by activating mTOR and its downstream effectors. The kinase mTOR interacts with several proteins to form two complexes mTORC1 and mTORC2 that leads to protein synthesis. AKT blocks key mediators of skeletal muscle atrophy transcriptional factor and ubiquitin ligases, thereby inhibiting nuclear translocation of the FoxO. Once phosphorylated by AKT, the FoxOs are excluded from the nucleus, and upregulation of MuRF1 and MAFbx is blocked so it leads to inhibiting protein degradation. Inhibition FoxO also leads to expression autophagy-related genes such as LC3 and thereby the autophagy process also decreased by inhibition of FoxO. The IGF1-AKT-mTOR pathway plays crucial role in muscle mass and function maintenance and also among elderly and can be also one of the potential targets of sarcopenia treatment. Macelignan found in nutmeg extract is already known to have PPAR-*γ* agonist effect. From previous in vitro study, nutmeg extract shows positive effects toward insulin sensitivity and glucose metabolism. Nutmeg extract may also have another activity that modulates phosphatidylinositol 3-kinase (PI3K) that can induce skeletal muscle hypertrophy or at least preserve muscle mass through protein synthesis via IGF1-AKT-mTOR pathway.

The fundamental role of satellite cells, IGF1-AKT-mTOR pathway for protein synthesis, and autophagy in muscle growth results in some alternative effort to find the substance or physical exercise that stimulates these factors as potent management for decrease in muscle mass due to aging. Skeletal muscle function and mass directly or indirectly correlated with homeostasis of autophagy activity in skeletal muscle. In our model, we are proposing that nutmeg may increase or at least prevent skeletal muscle mass loss during aging, and it is partly by inhibiting of autophagy activity in skeletal muscle (Figure 5).

## 5. Conclusion

Taken together, nutmeg extract free safrole and myristicin may increase muscle mass in aging rats partly via inducing AKT-mTOR-autophagy pathway. There is still open possibility in the phenomenon observed in our data whether these results especially in soleus muscle are caused by an increasing of muscle mass or preventing decrease muscle mass during aging. This potential nutmeg effect might become an alternative supplement for sarcopenia during aging.

## Data Availability

The data used to support the findings of this study are available from the corresponding author upon request.

## Supplementary Materials

Supplementary Materials Supplementary Table 1: primer sequences, annealing temperatures, and amplification cycles for semiquantitative Polymerase Chain Reaction (PCR). Supplementary Figure 1: photograph of age rat (80 weeks) body weight roughly around 450 gr compared with young rat (8 weeks) body weight roughly around 200-250 gr.
