# Supplementary material for: Nutmeg Extract Increases Skeletal Muscle Mass in Aging Rats Partly via IGF1-AKT-mTOR Pathway and Inhibition of Autophagy
Source: Evid Based Complement Alternat Med. 2018 Dec 17;2018:2810840. doi: 10.1155/2018/2810840 (PMC6311876; doi:10.1155/2018/2810840)
Supplement: Supplementary Materials — Supplementary Table 1: primer sequences, annealing temperatures, and amplification cycles for semiquantitative Polymerase Chain Reaction (PCR). Supplementary Figure 1: photograph of age rat (80 weeks) body weight roughly around 450 gr compared with young rat (8 weeks) body weight roughly around 200-250 gr. [file 2810840.f1.zip › 2810840.f1/Supplementary Figure Final 2018YN-RNY_ECAM_2601419.docx]

**Supplementary Figure 1**. Photograph of age rat Vs. young rat.

**Supplementary Figure 1**. Photograph of age rat (80 weeks) body weight roughly around 450 gr compared with young rat (8 weeks) body weight roughly around 200-250 gr.
