# Supplementary material for: Nutmeg Extract Increases Skeletal Muscle Mass in Aging Rats Partly via IGF1-AKT-mTOR Pathway and Inhibition of Autophagy
Source: Evid Based Complement Alternat Med. 2018 Dec 17;2018:2810840. doi: 10.1155/2018/2810840 (PMC6311876; doi:10.1155/2018/2810840)
Supplement: Supplementary Materials — Supplementary Table 1: primer sequences, annealing temperatures, and amplification cycles for semiquantitative Polymerase Chain Reaction (PCR). Supplementary Figure 1: photograph of age rat (80 weeks) body weight roughly around 450 gr compared with young rat (8 weeks) body weight roughly around 200-250 gr. [file 2810840.f1.zip › 2810840.f1/Table Final 2018 YN-RNY_ECAM_2601414.docx]

**Supplementary Table 1.** Polymerase Chain Reaction (PCR) Conditions

| **No** | **Primer** | **Gen Code**  **(Pubmed)** | | **Sequences** | **Tm (C)** | | **Time (sec)** | | **Cycles** | **Product**  **(bp)** |
| --- | --- | --- | --- | --- | --- | --- | --- | --- | --- | --- |
| 1 | ß-actin | [NM_031144.3](https://www.ncbi.nlm.nih.gov/entrez/viewer.fcgi?db=nucleotide&id=402744873) | **F** | **TGG AGA AGA TTT GGC ACC A** | | 60 | | 40 | 35 | 193 |
|  |  |  | **R** | **CCA GAG GCA TAC AGG GAC AA** | |  |  |  |  |  |
| 2 | IGF1 | [XM_006241193.3](https://www.ncbi.nlm.nih.gov/entrez/viewer.fcgi?db=nucleotide&id=1046894988) | **F** | **GCA TTG TGG ATG AGT GTT GC** | | 57 | | 20 | 35 | 202 |
|  |  |  | **R** | **GGC TCC TCC TAC ATT CTG TA** | |  |  |  |  |  |
| 3 | MHC 1 | [NM_017240.2](https://www.ncbi.nlm.nih.gov/entrez/viewer.fcgi?db=nucleotide&id=451958092) | **F** | **TTG CTC TAC CCA ACC CTA AGG ATG** | | 57 | | 20 | 35 | 81 |
|  |  |  | **R** | **TTG TGT TTC TGC CTG AAG GTG C** | |  |  |  |  |  |
| 4 | MyoD | [NM_176079.1](https://www.ncbi.nlm.nih.gov/entrez/viewer.fcgi?db=nucleotide&id=28570183) | **F** | **CGA CTG CCT GTC CAG CAT AG** | | 57,5 | | 40 | 34 | 174 |
|  |  |  | **R** | **GGA CAC TGA GGG GTG GAG TC** | |  |  |  |  |  |
| 5 | Myogenin | [NM_017115.2](https://www.ncbi.nlm.nih.gov/entrez/viewer.fcgi?db=nucleotide&id=21071081) | **F** | **TGA ATG CAA CTC CCA CAG C** | | 57 | | 20 | 35 | 164 |
|  |  |  | **R** | **CAG ACA TAT CCT CCA CCG TG** | |  |  |  |  |  |
| 6 | Pax 7 | [NM_001191984.1](https://www.ncbi.nlm.nih.gov/entrez/viewer.fcgi?db=nucleotide&id=300795245) | **F** | **AGC CGA GTG CTC AGA ATC AA** | | 60 | | 40 | 32 | 247 |
|  |  |  | **R** | **TCC TCT CGA AAG CCT TCT CC** | |  |  |  |  |  |

**Supplementary Table 1.** Primer sequences, annealing temperatures and amplification cycles for semi quantitative Polymerase Chain Reaction (PCR).
